# Discovery of Small Molecules Targeting Norovirus 3CL Protease by Multi-Stage Virtual Screening

**DOI:** 10.3390/ijms26125625

**Published:** 2025-06-12

**Authors:** Zhongling Shi, Na Liu, Fabao Zhao, Dongwei Kang, Steven De Jonghe, Johan Neyts, Ni Gao, Xinyong Liu

**Affiliations:** 1 Department of Medicinal Chemistry, Shandong Key Laboratory of Druggability Optimization and Evaluation for Lead Compounds, School of Pharmaceutical Sciences, Cheeloo College of Medicine, Shandong University, 44 West Culture Road, Jinan 250012, China; 2 Molecular, Structural and Translational Virology Research Group, Department of Microbiology, Immunology and Transplantation, Rega Institute for Medical Research, KU Leuven, Herestraat 49, B-3000 Leuven, Belgium; steven.dejonghe@kuleuven.be; 3 Antiviral Drug & Vaccine Research Group, Department of Microbiology, Immunology and Transplantation, Rega Institute for Medical Research, KU Leuven, Herestraat 49, B-3000 Leuven, Belgium; johan.neyts@kuleuven.be

**Keywords:** norovirus, 3C-like protease inhibitors, multi-stage virtual screening, MD simulation

## Abstract

Human noroviruses (HuNoVs) are the primary cause of acute viral gastroenteritis. There are no antivirals or vaccines available to treat and/or prevent HuNoV. Norovirus 3C-like protease (3CLpro) is essential for viral replication; consequently, the inhibition of this enzyme is a fruitful avenue for antinorovirus therapeutics. To discover novel 3CLpro inhibitors with diverse scaffolds, a multi-stage virtual screening approach was performed by docking >10 million compounds into the 3CLpro catalytic site. An initial subset of 18 compounds was selected, and compounds **YY-1029** and **YY-4204** were identified as the best two molecules. Molecular dynamics (MD) simulations and binding free energy calculations (MM/GBSA) of **YY-1029** and **YY-4204** were performed to elucidate the binding mechanisms. The ADMET properties were also estimated to predict the potential druggability of representative molecules. All 18 compounds were evaluated for their antinorovirus activity and cytotoxicity in a cell-based replicon system. This work could provide information for the development of 3CL pro inhibitors.

## 1. Introduction

Human noroviruses are the primary cause of sporadic and epidemic acute gastroenteritis worldwide. It is estimated that there are 685 million noroviruses cases per year, including 200 million cases amongst children under 5 years old. Morbidity is particularly high among the young and elderly, as well as immunodeficient individuals, with an estimated 200,000 deaths annually, including 50,000 child deaths. The burden of norovirus primarily impacts low-income countries. As a result of healthcare costs and economic losses, norovirus infection has been estimated to cost USD 60 billion globally [1,2]. Norovirus outbreaks pose significant challenges for containment even with routine sanitation practices. Notably, even the implementation of rigorous sanitary measures often falls short of preventing subsequent outbreaks. Compounding this issue is the current absence of effective vaccines, specific therapeutics, or prophylactics for norovirus, which together make the management of norovirus infections a formidable task.

Human noroviruses are positive-sense single-stranded RNA viruses which belong to the *Caliciviridae* family, specifically to genus Norovirus [3,4]. There are ten different genogroups (GI-GX), and GI, GII, and GIV are known to infect humans [5,6]. The norovirus genome (7–8 kb) consists of three open reading frames (ORF1-3), which encode a polyprotein (ORF1), a major capsid protein VP1 (ORF2), and a small basic protein VP2 (ORF3) [7]. The mature polyprotein precursor is processed by a virus-encoded 3C-like protease (3CLpro) to generate six mature nonstructural proteins (NS1-6) with distinct functions [6,8], including the formation of the membrane-associated replication complex ([NS1/2] p48, [NS3] p41, [NS4] p22), the initiation of genome translation ([NS5] VPg), the cleavage of the polyprotein ([NS6] protease), and the replication of the viral genome ([NS7] RdRp) [9]. Therefore, the co- and post-translational processing of the polyprotein by 3CLpro is essential for virus replication [10], which indicates that 3CLpro is a potential druggable target that could be used to develop small molecule inhibitors [11,12,13,14,15].

**Figure 1:**
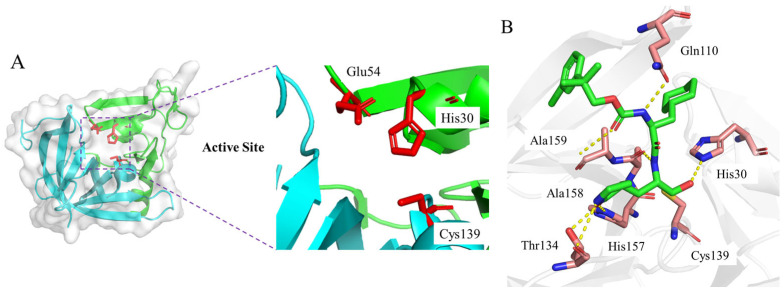
(**A**) Surface view of the 3CLpro crystal structure (PDB: 2FYQ) and active site with catalytic residues Cys139, His30, and Glu54 (red sticks). (**B**) X-ray crystal structure (PDB: 6W5H) of inhibitor **5d** (green sticks) with 3CLpro and hydrogen bond interactions with around residues (pink sticks).

Norovirus 3CLpro forms a typical chymotrypsin-like fold and contains the catalytic residues of Cys139, His30, and Glu54, and is located at the cleft between the N-terminal domain and the C-terminal domain (Figure 1A) [16]. The mechanism of action of norovirus 3CLpro is similar to related cysteine proteases, where Cys139 acts as a nucleophile, His30 functions as a general acid/base, and Glu54 facilitates the alignment of His30 and promotes the deprotonation of Cys139. The oxyanion of the tetrahedral intermediate is stabilized by the presence of an oxyanion hole adjacent to the 3CLpro active site [16,17]. Specifically, the X-ray crystal structure of inhibitor **5d** with 3CLpro elucidates that the aldehyde warhead is covalently bonded with Cys139 and a tetrahedral adduct is further stabilized by a hydrogen bond with His30 (Figure 1B) [13,14]. In addition, direct hydrogen bond interactions with Thr134, His 157, Ala 158, Ala 159, and Gln110 are also observed. These residues are highly conserved across norovirus proteases, and the mutation of these residues abolishes protease activity. Therefore, the active site is a desirable target region for discovering novel inhibitors.

Virtual screening is powerful enough to discover novel molecules with different scaffolds [18,19], which can mitigate scale, time, and cost issues compared to conventional experimental approaches [20,21,22]. In this paper, a multi-stage virtual screening strategy targeting the catalytic site of 3CLpro was applied to discover novel chemotypes as 3CLpro inhibitors. In total, 18 compounds were identified. Moreover, molecular dynamics (MD) simulations and MM/GBSA calculations were performed to investigate the binding mechanisms of representative molecules. The ADMET profiles of these molecules were also estimated to predict the potential druggability. Then, the antiviral activity and cytotoxicity of all the target compounds were assayed in a cell-based replicon system.

## 2. Results and Discussion

### 2.1. Multi-Stage Virtual Screening

**Figure 2:**
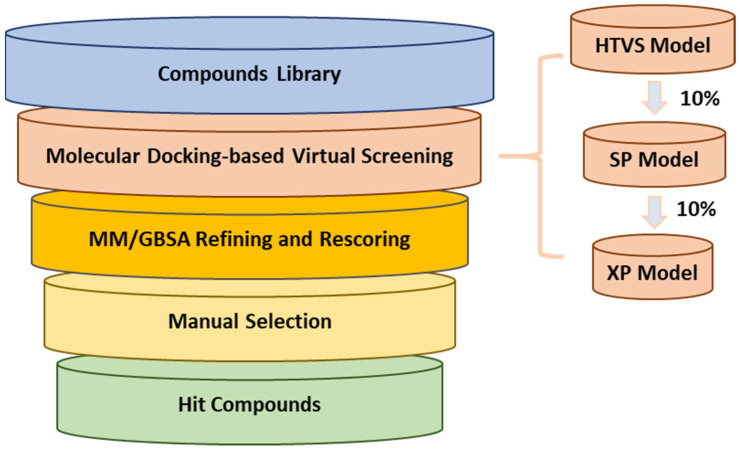
Flow chart of molecular docking-based virtual screening for discovery of 3CLpro inhibitors.

To discover small molecules as 3CLpro inhibitors, we adopted a multi-stage virtual screening strategy (Figure 2) targeting the active site of 3CLpro, including a molecular docking virtual screen and molecular mechanics/generalized born surface area (MM/GBSA) refinement and rescoring. The crystal structure of the catalytic domain of 3CLpro with inhibitor **5d** (PDB: 6W5H) was selected as the structure complex [13]. Four databases (Chembridge, Chemdiv, Enamine, and Vitas) were used. Molecular docking was performed at three different levels of precision in sequence, which enriched the data at every level, and only one-order-of-magnitude-fewer compounds needed to be studied at the next accuracy level higher. The high-throughput virtual screening (HTVS) docking precision mode efficiently enriched million-compound libraries, while the standard precision (SP) mode reliably performed high-accuracy docking for hundreds of thousands of ligands. The more accurate extra precision (XP) mode further eliminated false positives through expanded sampling and advanced scoring, resulting in even higher enrichment. The top 10% of best-scoring ligands for each procedure were selected for the next screening step, and about 6827 ligands were identified.

As we know, MM/GBSA is one of the most popular methods of estimating binding free energies, and can achieve a good balance between accuracy and computational efficiency, being more accurate than most scoring functions [23,24]. Therefore, 6827 top-ranked compounds were submitted to minimization-based MM/GBSA refinement and rescoring. Then, 1000 top-ranked binding poses were retained for further manual selection.

Due to the inherent inaccuracies of molecular docking, the visual inspection of binding modes remains a critical routine in the decision-making process. According to the criteria reported by Fischer et al. [25], we analyzed the 1000 top-ranked molecules by visual inspection and filtered based on binding conformation, shape complementarity, fundamental interactions with key residues and structural novelty, etc. Ultimately, 11 compounds from Vitas library, 6 compounds from the chembridge library, and 1 compound from the Chemdiv library were selected (Table 1).

From Table 1, we can see that these selected compounds showed desirable binding affinity (docking score) and binding free energies (MM/GBSA). None of these structures have been reported as Norovirus 3CL protease inhibitors. The docking scores of these compounds ranged from −8.761 to −10.474 kcal/mol, and the MM/GBSA results ranged from −49.24 to −74.98 kcal/mol. Specially, compound **YY-4204** possessed a remarkable docking score (−10.474 kcal/mol) and proper MM/GBSA data (−59.22 kcal/mol). Compound **YY-1029** possessed a promising docking score (−9.787 kcal/mol) and promising MM/GBSA data (−74.98 kcal/mol). According to the virtual screening results and structural novelty, compounds **YY-1029** and **YY-4204** were selected for further in silico study to elucidate dynamic behaviors and pharmacokinetics in detail.

### 2.2. Molecular Dynamics Simulations

**Figure 3:**
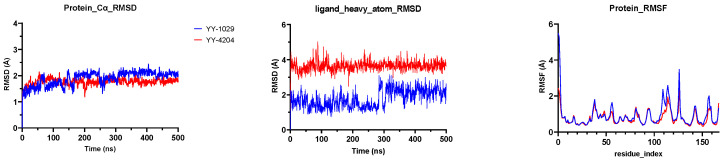
The RMSD and RMSF plots of **YY-1029** (blue line) and **YY-4204** (red line) binding to 3CLpro generated via MD simulations. Complete data can be found in the Supplementary Materials.

To study the stability and dynamic behaviors of these 18 molecules with the catalytic pocket, two representative molecules, **YY-1029** and **YY-4204,** were selected for molecular dynamics (MD) simulations (500 ns). The results showed that both **YY-1029** and **YY-4204** were located at the catalytic site of 3CLpro. The Root Mean Square Deviation (RMSD) is commonly used to assess the conformational drift of protein–ligand complexes by measuring the average change in displacement of a selection of atoms for a particular frame with respect to a reference frame [26]. As depicted in Figure 3, the RMSD plots showed that all 3CLpro-ligand complexes remained stable during the 500 ns simulation process, indicating that the overall structure of the 3CLpro did not change significantly due to the binding of the **YY-1029** (or **YY-4204**). Specifically, the protein-Cα RMSD showed that the coordinates of **YY-1029** (or **YY-4204**) fluctuated less than 2.5 Å in each 3CLpro-ligand complex and consistently bound stably to the 3CLpro active site. The ligand heavy atom RMSD of **YY-1029** was around 2.0 Å, which was around 4.0 Å for **YY-4204**. The observed values of proteins and ligands were closer, which indicated that the ligands did not diffuse away from the initial binding site. The Root Mean Square Fluctuation (RMSF, Figure 3) of 3CLpro-ligand were further investigated to characterize the local deviation of the protein chain [27].

**Figure 4:**
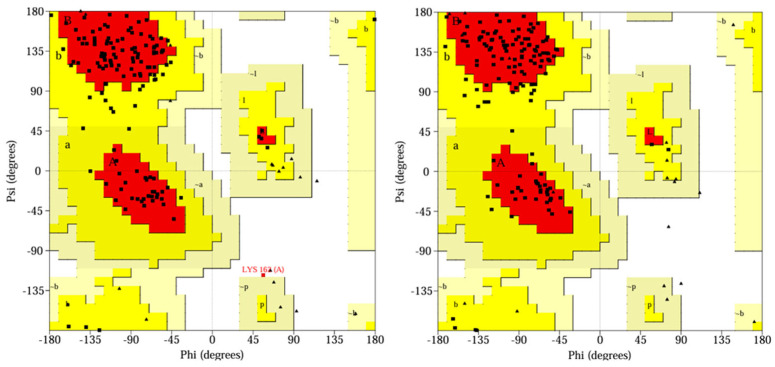
Ramachandran plots of **YY-1029**/3CLpro complex and **YY-4204**/3CLpro complex after 500 ns MD simulations. The Ramachandran plots provide a stereochemical assessment of the protein backbone conformation by displaying the distribution of φ (phi) and ψ (psi) dihedral angles for each residue. Squares represent non-glycine, non-proline residues, while triangles indicate glycine residues, which have greater conformational flexibility. The labeled regions—such as A, B, L for most favored, a, b, l, p for additionally allowed, and ~a, ~b, ~l, ~p for generously allowed—are derived from statistical analysis of high-resolution protein structures. The color intensity reflects the likelihood of conformational favorability, with darker areas indicating more favorable regions.

In addition, Ramachandran plots (Figure 4) showed that all 3CLpro residues were located in the most favored (more than 90%), additionally allowed, and generously allowed regions.

**Figure 5:**
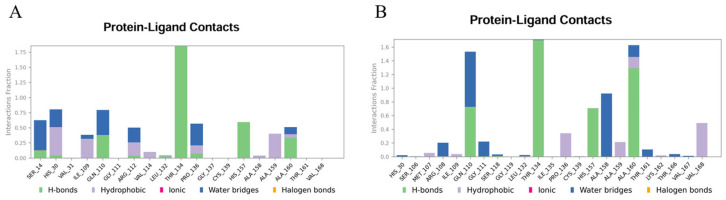
Interaction fraction plots of specific amino acid residues with **YY-1029** (**A**) and **YY-4204** (**B**).

**Figure 6:**
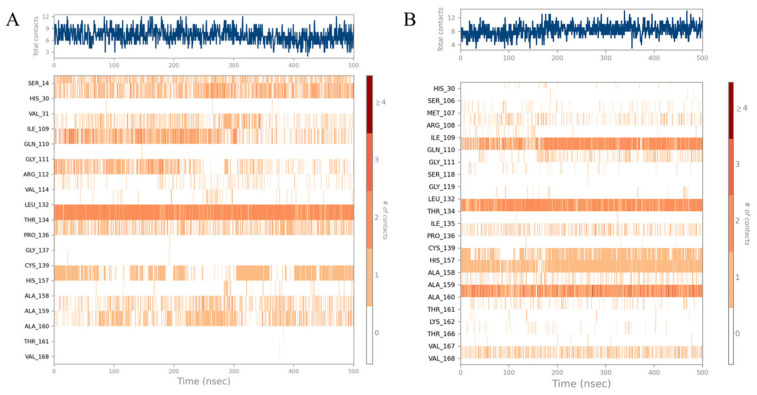
Number of protein–ligand contacts in each trajectory frame for 3CLpro with **YY-1029** (**A**) and **YY-4204** (**B**).

**Figure 7:**
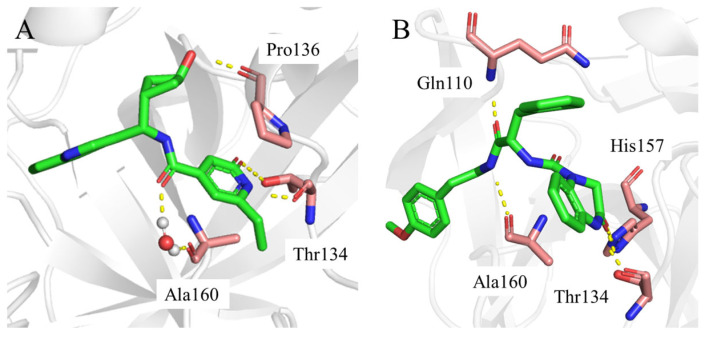
Binding modes of **YY-1029** (**A**) and **YY-4204** (**B**) generated from the MD simulation trajectory. Ligand: green stick; key residues: pink stick; salt bridge/hydrogen bond: yellow dashed line. The structure files (PDB files) can be found in the Supplementary Materials.

The MD simulation trajectories were clustered using the trajectory clustering program, and the most abundant clusters of 3CLpro/**YY-1029** complex and 3CLpro/**YY-4204** were extracted and are shown in Figure 5, Figure 6 and Figure 7. The detailed interactions between molecules and specific residues mainly included hydrogen bonds, hydrophobic contact, and water bridging. Notably, the interaction fraction plot (Figure 5) implied that the specific interaction remains constant over a certain percent of the 500 ns simulations. A value of 0.7 suggested that 70% of the simulation time, the specific interaction was maintained. Values over 1.0 were possible, as some residues may have made multiple contacts of same subtype with the ligand. In addition, Figure 6 visually represents the number of contact forces between **YY-1029** (or **YY-4204**) and 3CLpro in each trajectory frame, as well as the specific residue codes that interacted with the ligand, with darker orange reflecting stronger interactions.

Consistent with the results in Figure 5 and Figure 6, the two-dimensional ligand interaction diagrams demonstrated that ligands established signature hydrogen bonds with residues of the catalytic site, which contributed to the critical role of maintaining the protein–ligand binding affinity. **YY-1029** (Figure 7A) formed hydrogen bonds with residues Thr134 and Pro136, and water bridges with residue Ala160. **YY-4204** (Figure 7B) formed multiple interactions with residues Thr134, His157, Gln110, and Ala160.

Overall, the MD simulation results indicated that the complexes of **YY-1029** (or **YY-4204**) with 3CLpro maintained stable conformations and extensive protein–ligand interactions, and the Ramachandran plots validated the structural stability of all 3CLpro/**YY-1029** (or **YY-4204**) complexes, contributing to the high binding affinity between molecules and 3CLpro.

### 2.3. Binding Free Energy Calculations

The binding free energy (ΔG_Bind_) of **YY-1029** (−328.61 kcal/mol) and **YY-4204** (−328.82 kcal/mol) with 3CLpro was calculated using MM/GBSA (Table 2). The ΔG_vdW_ value of these two compounds accounted for the most part for ligand binding free energy (ΔG_Bind_). That is, the binding affinity mainly benefited from the Van der Waals interaction between the ligand and residues of the catalytic site.

### 2.4. ADMET Properties Prediction

The main reasons for the failure of drug development are undesirable pharmacokinetics and higher toxicity. It is widely recognized that the absorption, distribution, metabolism, excretion, and toxicity (ADMET) of chemicals should be evaluated as early as possible [28,29,30]. Therefore, the ADMET profiles of these 18 representative molecules were predicted to guide subsequent structural optimization and preliminary drug-like property evaluations. ADMETlab 3.0 provides comprehensive evaluations of ADMET properties, as well as some physicochemical properties and medicinal chemistry friendliness [31]. ProTox 3.0 provides predictions of oral toxicity [32].

**Figure 8:**
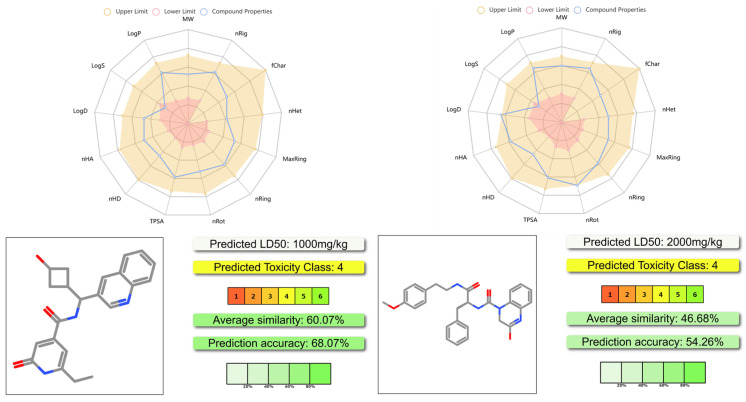
Physicochemical properties and toxicity prediction results of **YY-1029** and **YY-4204** using ADMETlab 3.0 and Pro-Tox 3.0 server. Complete data can be found in Supplementary Materials.

The results are shown in Figure 8 and the supporting information. As shown in Figure 8, both **YY-1029** and **YY-4204** had proper physicochemical properties, acceptable ADMET profiles, and lower toxicity. The predicted LD_50_ of **YY-1029** and **YY-4204** was 1000 mg/kg and 2000 mg/kg, respectively. The results suggested that these two molecules achieved a good balance between moderate efficacy and safety profiles.

### 2.5. Biological Evaluation

The purchased compounds were evaluated for their anti-norovirus activity in a cell-based replicon system. The EC_50_ and CC_50_ values are listed in Table 3. Unfortunately, no compounds showed antiviral activity at a concentration of 50 µM. Meanwhile, all compounds showed weak cytotoxicity (CC_50_ > 50 µM) except for **YY-9837**.

### 2.6. Discussion

We employed a multi-stage virtual screening strategy targeting the essential norovirus 3CL protease, successfully identifying 18 diverse small molecule scaffolds predicted to bind potently within the enzyme’s catalytic site based on docking scores and MM/GBSA binding free energy calculations. Molecular dynamics simulations further demonstrated stable binding for representative hits, YY-1029, and YY-4204, alongside acceptable predicted ADMET profiles, suggesting potential draggability. Despite these promising in silico characteristics, all compounds disappointingly exhibited negligible antiviral activity (EC_50_ > 50 µM) in cell-based antiviral assays. This significant gap between predicted target engagement and observed cellular efficacy echoes challenges frequently reported in recent norovirus drug discovery efforts. Similar discrepancies have been noted by others, such as Rathnayake et al. (2020), who found that computationally identified 3CLpro inhibitors often required substantial optimization to achieve cellular activity, primarily due to bioavailability limitations [13]. Wang et al. (2022) also highlighted the difficulty non-covalent binders face in overcoming cellular permeability barriers, suggesting this is a common hurdle inherent to many in silico-derived hits targeting intracellular enzymes [17]. Our findings thus reinforce the understanding that achieving sufficient intracellular concentrations of inhibitors remains a critical bottleneck in translating computational predictions into effective antivirals.

The observed lack of activity underscores the multifaceted nature of drug discovery. While our computational models focused effectively on target binding, they could not fully account for crucial biological barriers such as cellular membrane permeability, potential metabolic instability, the complex intracellular environment where the viral replication complex resides, or off-target effects (as suggested by the isolated cytotoxicity of YY-9837). Furthermore, while MM/GBSA provides valuable ranking insights, its approximations regarding solvation, entropy, and protein dynamics can overestimate absolute binding affinity, explaining the stark contrast between calculated ΔG values of approximately −328 kcal/mol and the absence of measurable cellular potency. These limitations are inherent to purely computational approaches necessitate experimental validation and focused optimization.

Moving forward, our efforts will shift towards bridging this gap through the targeted hit optimization of the identified series. Future research will prioritize doing the following: firstly, experimentally determining cellular permeability and metabolic stability to pinpoint key liabilities; secondly, pursuing structural biology efforts via X-ray crystallography to obtain definitive complexes of inactive hits (e.g., YY-1029) bound to 3CLpro, allowing for a critical comparison with our MD-predicted poses and revealing precise structural insights for rational design; and thirdly, employing structure-guided medicinal chemistry strategies. This chemistry optimization will explicitly aim to enhance cellular bioavailability while preserving target engagement. Specific tactics may include introducing cell-penetrating groups, modulating lipophilicity (logP) towards optimal ranges (e.g., 1–3), exploring prodrug strategies similar to successful approaches for other viral protease inhibitors [12,14], and addressing potential off-target interactions identified through counter-screening. Concurrently, we plan to implement more sophisticated computational approaches, such as ensemble docking, to capture the dynamic flexibility of the target that may be missed using static structures.

In conclusion, while this virtual screening campaign successfully identified novel chemical starting points interacting with the norovirus 3CLpro active site, the lack of cellular activity emphasizes the crucial need to address the challenges of intracellular delivery and target accessibility in subsequent optimization cycles. Our findings align with the recent literature highlighting bioavailability as a central translational hurdle in norovirus inhibitor development. By integrating detailed structural insights gained from experimental complex determination with focused medicinal chemistry aimed at improving permeability and stability, we aim to transform these initial inactive computational hits into leads worthy of further evaluation, advancing the crucial pursuit of effective therapeutics against norovirus infections. The structural data and identified chemotypes generated here provide a valuable foundation for these future efforts.

## 3. Materials and Methods

### 3.1. Target Protein Structure Preparation

The co-crystal complex structure of 3CLpro with ligand (PDB: 6W5H) was downloaded from the protein data bank (PDB, rcsb.org) and used as a target protein for virtual screening. The protein preparation wizard (Schrödinger Suite 2023-3) was used to prepare the structure. The co-crystalized factors (such as ion, metals, and non-water solvents) were removed, and water molecules beyond 8 Å of the binding ligand were deleted. The protein hydrogen atoms were also deleted and re-added. The terminal oxygen atoms and missing side chains were added, and mislabeled elements were corrected with the pH = 7.4 ± 0.2. Bond orders were assigned using the Chemical Component Dictionary (CCD), and hydrogen bonds were optimized by PROPKA. The energy minimization was restrained using the OPLS4 force field. The other parameters were set to default.

### 3.2. Target Protein Grid Generation

The prepared protein was considered for the grid generation, and a 3-dimensional boundary for the ligand binding was generated using the “Receptor Grid Generation” panel of the Glide module (Schrödinger Suite 2023-3). The size of the receptor grid was set to default. All other parameters were set to default.

### 3.3. Ligand Preparation

More than 10 million commercially available compounds from four vendors (Chembridge, Chemdiv, Enamine, and Vitas) were used for screening. The PAINS structures were filtered before the screening. All compounds for docking applied the OPLS4 force field to optimize the structures using the LigPrep module (Schrödinger Suite 2023-3). Two-dimensional structures in .sdf format were downloaded from the libraries, which were then prepared into 3D structures. The ionization state of all ligands was optimized with pH = 7.0 ± 0.5 using Epik, which is based upon the Hammett and Taft methodologies. The torsional bond of ligands was released, and a maximum of one stereoisomer per ligand was generated. The other parameters were set to default with the OPLS4 force field.

### 3.4. Multi-Stage Virtual Screening Method

Virtual screening was performed using the Glide program (Schrödinger Suite 2023-3). Ligand-flexible docking was performed using the prepared libraries of compounds and the virtual screening workflow protocol of Glide. The protein residues were set as rigid to balance efficiency and accuracy, and all other parameters were set to default. The software internally generated different conformations, which passed across several filters viz. Euler angles, grid-based force field evaluation, and Monte Carlo energy minimization. Lastly, the evaluation of conformers took place based on the docking score, and one of the best conformations per ligand was generated as an output. There was a rational workflow for virtual screening from HTVS to SP to XP at three different levels of precision docking in sequence. HTVS and SP utilized the self-same scoring function, whereas XP reduced the intermediate conformations and thoroughness of the torsional refinement and sampling. A total of 10 million compounds were docked against 3CLpro. Subsequently, top scorers were set forth for SP docking, and the output of SP docking was put forward in XP docking. The docking score is a binding energy/affinity given in the kcal/mol. The top 10% of best-scoring ligands for each procedure were selected for the next screening step, and about 6827 ligands were identified.

Further, these ligands were rescored based on binding energy using the Prime MM/GBSA module (Schrödinger Suite 2023-3). The residues within 5 Å of the ligands were set to be flexible. The binding poses of the top 1000 ranking ligands were checked manually, and 18 target compounds were selected based on the docking scores, MM/GBSA scores, and manual selection (Table 1).

### 3.5. MD Simulations and Binding Free Energy Calculations

All-atom MD simulations were performed using the Desmond module (Schrödinger suite 2023-3) as implemented in Maestro. The initial docking poses were used to build the simulation system. The atomic framework was solvated with a TIP3P water model with orthorhombic intermittent limit conditions for a 10 Å buffer region. The overlapping water molecules were eliminated. Na^+^ or Cl^−^ was added as counter ions to neutralize the systems. The particle mesh Ewald method was used to calculate the long-range electrostatic interactions. A cut-of radius of 9.0 Å was applied for short-range van der Waals and Coulomb interactions. Each solvated system was minimized and equilibrated using the default protocol of Desmond, which included 2 NVT and 2 NPT restrained short simulations. A temperature of 300 K and a pressure of 1 atm was maintained in the systems using the Nosè–Hoover chain thermostat and Martyna–Tobiase–Klein barostat methods, respectively. A hybrid energy minimization algorithm with 1000 steps of steepest descent, followed by conjugate gradient algorithms, was used. All equilibrated systems were then submitted to an MD simulation run with periodic boundary conditions in the NPT ensemble using OPLS4 force field for 500 ns. The post-dynamics simulations were analyzed using the simulation interaction diagram module. The binding energy between the 3CLpro and the docked ligands was calculated over the 500 ns period using the MM/GBSA module based on the MD simulations. It takes a Desmond trajectory file, splits it into individual snapshots, runs the Prime-MMGBSA calculations on each frame (5 ns), and yields the average calculated binding energy. Details of the starting systems for MD simulations and MM/GBSA date can be found in the Supplementary Materials.

### 3.6. ADMET Properties Predication

The compounds’ ADMET properties were estimated using ADMETlab 3.0 (https://admetlab3.scbdd.com/server/evaluation, accessed on 25 November 2024) and ProTox 3.0 (https://tox.charite.de/protox3/index.php?site=compound_input, accessed 25 November 2024). The molecular SIMLE strings were generated using Chemdraw and then submitted to the online systems. Analysis reports were then generated, which can be found in the Supplementary Materials.

### 3.7. Biological Evaluation Method

#### 3.7.1. Viruses and Cells

Murine norovirus (MNV, strain MNV-1. CW1) was propagated as previously described [33]. HG23 cells and RAW 264.7 cells were maintained as described earlier [34,35]. The cells were incubated at 37 °C in a humidified atmosphere of 5% CO_2_. To obtain virus stock, once full CPE was observed, the cells underwent two freeze–thaw cycles and the virus was harvested from the supernatants after centrifugation (10 min, 1000× *g*) and stored at 80 °C. The viral titer was determined by endpoint titration.

#### 3.7.2. Compounds

The compound libraries were purchased from Chembridge, Chemdiv, and Vitas.

#### 3.7.3. Antiviral and Cytotoxicity Assay

The experiment was performed as previously described [35]. In short, RAW264.7 cells (1 × 10^4^ cells/well) were infected with MNV.CW1 in the presence of a dilution series of compounds. Antiviral activity and cytotoxicity were determined using a colorimetric assay using 3-(4,5-dimethylthiazol-2-yl)-5-(3-carboxymethoxyphenyl)-2-(4 sulfophenyl)-2H-tetrazolium (MTS). The 50% effective concentration (EC_50_) was defined as the compound concentration that protected 50% of the cells from CPE. The cell viability % was calculated as (OD_treated_/OD_CC_) × 10 and the 50% cytotoxic concentration (CC_50_) was defined as the compound concentration that reduces the number of viable cells by 50%. HuNoVGI.1replicon: The experiment was performed as described earlier with minor modifications. In short, HG23 cells (7.5 × 10^2^ cells/well) were seeded into the wells of a 96-well plate without G418 (Geneticin Selective Antibiotic). After 24 h of incubation, the tested compounds were added. The cells were incubated for another 72 h; then they were collected for RNA load quantification using RT-qPCR [34]. The EC_50_ values were defined as the compound concentration that led to a 50% reduction in the relative HuNoV GI.1 replicon RNA levels.

## 4. Conclusions

HuNoV infections are a significant worldwide health burden for all age groups, but particularly for the young, the elderly, and immunocompromised populations. No approved antiviral therapeutics are currently available for HuNoV infection. Robust post-infection therapeutics would be beneficial for these populations. HuNoV 3CLpro is a prime drug target, given its importance in mediating viral replication, and could be used for developing small-molecule inhibitors. Virtual screening is a powerful tool used to discover novel molecules with different scaffolds, which can mitigate scale, time, and cost issues. In this paper, we used a multi-stage virtual screening approach to discover 18 small molecules with diverse scaffolds. According to the docking scores and MM/GBSA results, the compounds **YY-1029** and **YY-4204** were identified as the best two molecules. MD simulations were performed to elucidate the binding mechanisms, and MM/GBSA calculations were performed to calculate the binding affinity of these two molecules. The ADMET properties of **YY-1029** and **YY-4204** were also estimated, demonstrating potential druggability. All 18 compounds were evaluated for their antinorovirus activity and cytotoxicity in a cell-based replicon system. As all compounds displayed low antiviral activity (EC_50_ > 50 µM) and weak cytotoxicity (CC_50_ > 50 µM), and further research work should be conducted to discover more potent antinorovirus inhibitors.

## Figures and Tables

**Table 1:** Library ID, chemical structures, docking score (kcal/mol), and MM/GBSA (kcal/mol) of compounds selected from virtual screening.

ID	Library ID	Chemical Structure	Docking Score	MM/GBSA
YY-0809	11130809	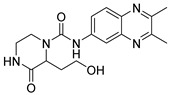	−9.955	−49.24
YY-1029	99071029	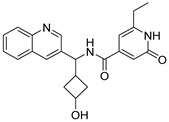	−9.787	−74.98
YY-0587	34940587	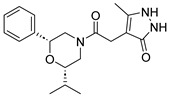	−9.451	−56.61
YY-4095	22844095	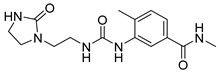	−9.344	−65.35
YY-0734	K279-0734	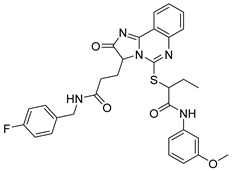	−9.812	−70.98
YY-8610	STK618610	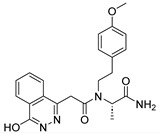	−9.755	−57.95
YY-4047	STL534047	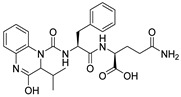	−9.754	−52.98
YY-9773	STK609773	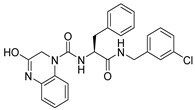	−9.588	−54.35
YY-6127	STK616127	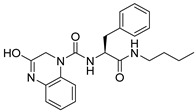	−9.413	−49.70
YY-4204	STK614204	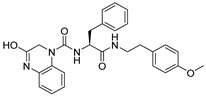	−10.474	−59.22
YY-9837	STK609837	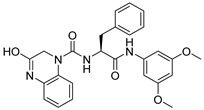	−9.343	−59.22
YY-3724	STK623724	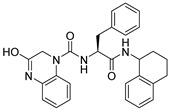	−9.336	−63.94
YY-9242	STK599242	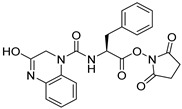	−8.987	−63.86
YY-0182	STL510182	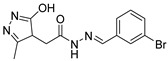	−9.306	−45.63
YY-3656	40203656	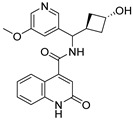	−8.761	−57.30
YY-0744	80583606	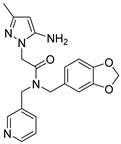	−9.728	−49.17
YY-4958	STL544958	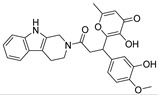	−9.475	−57.81
YY-0420	STL550420	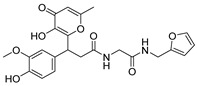	−8.842	−62.28

**Table 2:** MM/GBSA (kcal/mol) of **YY-1029** and **YY-4204** with 3CLpro ^a^.

ID	ΔG_Bind_	ΔG_Coulomb_	ΔG_Hbond_	ΔG_Lipo_	ΔG_vdW_
**YY-1029**	−328.61	−140.66	−11.94	−72.53	−252.99
**YY-4204**	−328.82	−115.56	−10.73	−77.13	−263.20

^a^ The whole simulation trajectory was sampled, and the estimation was calculated every 5 ns. The averages of all calculations are presented as the results. The complete dataset and standard deviations can be found in the Supplementary Materials.

**Table 3:** Cell-based activity (EC_50_) and cell cytotoxicity (CC_50_) values of compounds.

ID	EC_50_ (µM) ^a,b^	CC_50_ (µM) ^a,c^
YY-0809	>50	>50
YY-1029	>50	>50
YY-0587	>50	>50
YY-4095	>50	>50
YY-0734	>50	>50
YY-8610	>50	>50
YY-4047	>50	>50
YY-9773	>50	>50
YY-6127	>50	>50
YY-4204	>50	>50
YY-9837	>50	44.95
YY-3724	>50	>50
YY-9242	>50	>50
YY-0182	>50	>50
YY-3656	>50	>50
YY-0744	>50	>50
YY-4958	>50	>50
YY-0420	>50	>50

^a^ Data are mean values of two to three independent experiments, each one in triplicate. ^b^ CC_50_: cytotoxic concentration (μM) to induce 50% death of non-infected cells. ^c^ EC_50_: effective concentration (µM) to inhibit norovirus-induced cell death by 50%.

## Data Availability

The data presented in this study are available on request from the corresponding author. The raw data can be found at Zenodo with the following link: https://doi.org/10.5281/zenodo.15615849.

## Supplementary Materials

The following supporting information can be downloaded at https://www.mdpi.com/article/10.3390/ijms26125625/s1.
